# Amino acid substitutions in the H5N1 avian influenza haemagglutinin alter pH of fusion and receptor binding to promote a highly pathogenic phenotype in chickens

**DOI:** 10.1099/jgv.0.001672

**Published:** 2021-11-02

**Authors:** Joshua E. Sealy, Wendy A. Howard, Eleonora Molesti, Munir Iqbal, Nigel J. Temperton, Jill Banks, Marek J. Slomka, Wendy S. Barclay, Jason S. Long

**Affiliations:** ^1^​ Avian Influenza Group, The Pirbright Institute, Woking, GU24 0NF, UK; ^2^​ Virology Department, Animal and Plant Health Agency (APHA-Weybridge), Woodham Lane, Addlestone, Surrey KT15 3NB, UK; ^3^​ Viral Pseudotype Unit, Medway School of Pharmacy, University of Kent, UK; ^4^​ VisMederi Research S.r.l., Siena, Italy; ^5^​ Department of Infectious Disease, Faculty of Medicine, Imperial College London, St. Mary’s Campus, London W2 1NY, UK; ^6^​ Division of Virology, National Institute for Biological Standards and Control, Blanche Lane, South Mimms, Potters Bar EN6 3QG, UK

**Keywords:** avian influenza, H5N1, pathogenicity, haemagglutinin, influenza, fusion

## Abstract

Highly pathogenic H5N1 avian influenza viruses cause devastating outbreaks in farmed poultry with serious consequences for animal welfare and economic losses. Zoonotic infection of humans through close contact with H5N1 infected birds is often severe and fatal. England experienced an outbreak of H5N1 in turkeys in 1991 that led to thousands of farmed bird mortalities. Isolation of clonal populations of one such virus from this outbreak uncovered amino acid differences in the virus haemagglutinin (HA) gene whereby the different genotypes could be associated with distinct pathogenic outcomes in chickens; both low pathogenic (LP) and high pathogenic (HP) phenotypes could be observed despite all containing a multi-basic cleavage site (MBCS) in the HA gene. Using reverse genetics, three amino acid substitutions in HA were examined for their ability to affect pathogenesis in the chicken. Restoration of amino acid polymorphisms close to the receptor binding site that are commonly found in H5 viruses only partially improved viral fitness *in vitro* and *in vivo*. A third novel substitution in the fusion peptide, HA_2_G4R, enabled the HP phenotype. HA_2_G4R decreased the pH stability of HA and increased the pH of HA fusion. The substitutions close to the receptor binding site optimised receptor binding while modulating the pH of HA fusion. Importantly, this study revealed pathogenic determinants beyond the MBCS.

## Introduction

Avian influenza viruses (AIVs) that carry a highly pathogenic (HP) phenotype are thought to have caused disease outbreaks recorded in gallinaceous poultry since as early as 1878 [1, 2]. However, only in recent decades have HPAIVs such as the H5N1 subtype attracted concern as regards zoonotic infections because of their high case fatality rate and the persistent threat posed from infected chickens and ducks [3, 4]. Human infection caused by the ‘goose/Guangdong’ (GsGd) lineage H5N1 first occurred in 1997 in Hong Kong SAR followed by re-emergence in 2003 in mainland China, followed by subsequent endemicity in poultry in the Western Pacific and South-East Asian regions [5, 6]. Since then, these GsGd viruses have continued to cause zoonotic outbreaks, primarily in the Western Pacific and South-East Asian regions, but also spread through the European, Eastern Mediterranean, and African regions [7–10]. Following subsequent reassortment events affecting the neuraminidase (NA) and viral internal genes, currently circulating H5Nx viruses derived from the GsGd lineage have evolved into descendant clades and continue to be disseminated by wild birds with subsequent transmission risks to poultry and zoonotic concerns [3, 11–14].

One of the primary causes of the HP phenotype in AIVs is the acquisition of a multi-basic cleavage site (MBCS) in the haemagglutinin (HA) protein that facilitates systemic infection. The MBCS allows for HA to be proteolytically activated by ubiquitous intracellular furin-like serine proteases such as furin and PC6 [15–17]. Without this MBCS, precursor HAs would ordinarily be activated by trypsin-like proteases that are restricted to respiratory or intestinal epithelia [18]. Such viruses would typically carry a low pathogenic (LP) phenotype. However, the existence of an MBCS does not guarantee a HP phenotype. Indeed, experiments that artificially introduce an MBCS into H1, H3, H10 and H11 show these virus subtypes to be refractive to a HP phenotype [19, 20].

Early investigations into the phenotype of H5 viruses carrying an MBCS showed differences in pathogenesis between different avian hosts and various degrees of virulence and capacities to transmit by contact to naive birds [21]. In these initial investigations, ducks were shown to be resistant to infection, one H5N1 virus, A/chicken/Scotland/59 was shown to be pathogenic in chickens but not turkeys, and a H5N9 virus, A/turkey/Ontario/7732/66 was shown to be pathogenic in turkeys but not chickens [21]. H5N1 viruses with an HP phenotype in ducks have been isolated on several occasions and the amino acid residues shown to facilitate this HP phenotype have been shown to exist on multiple influenza genes, including HA, albeit at residues distal to the MBCS [22, 23]. Taken together, it is evident that the transition of an AIV from an LP to an HP phenotype is complex and involves several virus factors beyond the MBCS.

Prior to the emergence of the GsGd lineage, an H5N1 virus, namely A/turkey/England/50-92/1991 (50-92), was isolated in late 1991 from a devastating outbreak in farmed turkeys in England. The virus 50–92 was shown to have an intravenous pathogenicity index (IVPI) score of 3.0, confirming it to be an HPAIV [21]. Interestingly, plaque clones in MDCK cells were isolated and shown to have either an LP or an HP phenotype, and potential causative substitutions were identified in the HA gene [24]. The three amino acid residues that differed were at positions 160, 193 (H3 numbering used throughout) close to the receptor binding pocket and the fourth amino acid of HA2 (HA_2_4 or H5 aa residue 348) in the fusion peptide; both LP and HP viruses retained the MBCS. A second H5N1 virus (A/turkey/England/87-92/1991) was isolated from this outbreak and shown to have an IVPI of 0.00 [24]. This virus was similar to the 50–92 virus in that it shared the same amino acids at residues 193 and HA_2_4; it differed from 50 to 92 at amino acid residue 160. We hypothesised that the three substitutions present in the 50–92-HP clones (160T, 193K, HA_2_4R) in the HA gene were required for efficient HA binding, and fusion. In this study, we aimed to generate variants of the 50–92 virus at all three HA positions and elucidate the mechanism by which these amino acids can alter the pathogenesis of H5N1 virus 50–92 in the chicken host.

## Methods

### Cells and eggs

Chicken embryo fibroblast (CEF), human embryonic kidney (293T), chicken fibroblast (DF-1), Madin-Darby canine kidney (MDCK) and human epithelial (HeLa) cells were maintained in Dulbecco’s modified Eagle’s medium (DMEM; Invitrogen) supplemented with 10 % foetal calf serum (FCS) (Biosera) and 1 % penicillin-streptomycin (PS; 100× stock at 10 000 IU Penicillin and 10 000 µg ml^−1^ Streptomycin). The cell lines were maintained at 37 °C in a 5 % CO_2_ incubator. DF-1 cells were maintained at 39 °C. Cell lines were trypsinised and passaged twice weekly. CEF cells were generated from 9 to 10 day old chicken embryos using 0.25 % trypsin (Invitrogen) [25]. Virus was grown in 10 day old specific-pathogen-free (SPF) embryonated hens’ eggs which were monitored daily by candling. Eggs infected with HPAIV were monitored routinely and chilled ideally at the point where the integrity of egg veins were decreasing, and embryo viability was dropping. However, in some instances eggs were chilled when death had already occurred. LPAI infected eggs were chilled after 2 to 3 days-post-infection (dpi). Allantoic fluid was harvested using sterile technique and stored at −70 °C.

### Recombinant virus and site-directed mutagenesis

The reverse genetics system for generation of recombinant A/turkey/England/50-92/1991 (H5N1) (50-92) virus has been described previously [26]. Mutagenesis of the 50–92 HA gene was carried out sequentially to introduce each of the three HA substitutions and removal of the HA multi-basic cleavage site for sb50-92 viruses using the QuikChange Lightning Site-Directed Mutagenesis Kit. The 50–92, 50-92_160T_ 50-92_160T,193K_, 50-92_4R_ and 50–92-HP HA genes were successfully inserted into the pCAGGS expression vector via the introduction of the NotI and MluI restriction enzyme sites on the 5′ and 3′ ends by PCR. All plasmid constructs were verified by DNA sequencing and all virus HA substitutions were verified by sequencing the mutated region of the viral gene of successfully generated reverse-genetics (RG) virus.

### *In vitro* virus growth kinetics

Influenza viruses were first titrated by plaque assay; see section Visualisation of plaque morphology in MDCK cells. Plaques were counted and used to determine the plaque forming units per millilitre of virus. This was then used to achieve the desired multiplicity of infection (MOI). CEF cells in 6-well plate format were inoculated at a MOI of 10^−3^, washed once in PBS and incubated in 2 ml DMEM supplemented with 2 % FCS and 1 % PS at 37 °C. At the times indicated, cell supernatant was collected, vRNA isolated using the Qiaquick RNA extraction method (Qiagen) as previously described [27], and viral RNA determined by qRT-PCR. Briefly, viral RNA was quantified by influenza Matrix (M gene) qRT-PCR, as previously described [28]. Relative EID_50_ units (REU) were calculated by quantitative standards of five ten-fold dilutions of extracted RNA from 10^6^ EID_50_ A/turkey/Turkey/1/2005 [27]. Not detected (negative) samples were deemed so if they held a ct value of more than 35–36, depending on the ct values observed for the negative and positive controls included in the qRT-PCR run. For each qRT-PCR the negative control included the RNase-free water used to dilute the PCR reaction, and the positive control included vRNA at a known concentration that was extracted at the same time as the RNA isolation of the samples to be tested.

### Visualisation of plaque morphology in MDCK cells

Confluent monolayers of MDCK cells in 12-well plates were prepared for infection beforehand. Virus was diluted in SF DMEM in a ten-fold dilution series. Cell monolayers were washed with PBS, PBS was then removed and inoculated with 200 µl of virus and incubated at 37 °C for 1 h. Following inoculation, 1 ml of flu overlay and avicel (Sigma Aldrich) mix (2 : 1 ratio) with or without 1µg ml^−1^ TPCK trypsin (Sigma Aldrich) was added to each well [29]. After 3 days incubation at 37 °C, the overlay was removed, and cells fixed/stained using 0.1 % crystal violet solution (1 % crystal violet in water (Sigma-Aldrich Cat. No. HT901-8FOZ), 20 % methanol). Monolayers were washed before imaging using a flatbed scanner.

### Animal studies

For the IVPI tests, all procedures were conducted in accordance with OIE guidelines [30]. Briefly, ten 6-week-old White Leghorn chickens, hatched from eggs obtained from a SPF flock, were intravenously infected with a minimum of 16 haemagglutination units (HAU) of virus diluted one in ten in sterile PBS. Clinical signs were scored in accordance with the IVPI test and recorded daily for 10 days, after which, surviving birds were humanely killed. Oropharyngeal, cloacal and blood samples were taken from all chickens prior to infection to test for current and previous influenza infection. Samples revealed no current infection with H5N1 by M gene qRT-PCR and no previous exposure to H5N1 infection by haemagglutination inhibition antibody testing [30] using A/turkey/England/50-92/91 antigen (APHA, Weybridge).

### Split venus HA fusion assay

Confluent 6-well plates of cells were transfected with 1250 ng pCAGGS 50–92 HA or empty pCAGGs together with 1250 ng pBiFC-bJunVN155 or with 1250 ng of pBiFc-bFosVC155 alone [31]. Three hours after transfection, cell monolayers were washed in serum free DMEM and trypsinised before mixing the HA+pBiFC-bJunVN155 cells with the pBiFC-bFosVC155 cells in a 1 : 1 ratio and seeding into clear bottomed black sided 96-well plates (Corning). After 24 hours cells were washed with 100 µl pre-warmed serum free DMEM and treated with 100 µl MES pH buffer at the indicated pH at 37 °C for 5 min before cell media was replaced. After 3 h syncytia were imaged by fluorescence microscopy. Fluorescence intensity of cells was measured with a FLUOstar Omega plate reader (BMG Labtech).

### pH stability of 50-92 H5 pseudotyped virus like particles

The 50–92 HA pseudotyped viruses (PVs) encoding firefly luciferase were generated in HEK 293 T cells and treated with exogenous NA (Sigma Aldrich) as previously described [32, 33]. Then 50 µl of PV stock was mixed gently with an equal volume of pre-warmed MES pH buffer at the indicated pH in 1.5 ml Eppendorf tubes and incubated at 37 °C for 5 min before inoculation of 25 µl per well of confluent HEK 293 T cells in a white bottomed 96-well plate. After 24 hours cell media was removed, and cells were lysed in passive lysis buffer (Promega) before quantification using a firefly Luciferase Assay System (Promega) and a FLUOstar Omega plate reader (BMG Labtech).

### Biolayer interferometry

Viruses were propagated in embryonated hen’s eggs and concentrated by centrifugation at 135 000 ***g*** for 2 h at 4 °C. Concentrated virus was then resuspended in PBS and purified using a 30–60 % continuous sucrose gradient at 135 000 ***g*** for 2 h at 4 °C. Purified virus was recovered from the sucrose gradient and centrifuged a final time to form a pellet which was resuspended in PBS. Purified virus was then quantified by NP-ELISA. Virus was serially diluted two-fold in PBS and adsorbed to Immulon 2HB (Thermo: 10795026) plates overnight at 4 °C. Plates were then blocked with blocking buffer (5 % skimmed milk powder, 0.1 % Tween-20) for 1 h at room temperature, washed with washing buffer (0.1 % Tween-20) then viruses permeabilised with 0.2 % (v/v) Triton X-100 in PBS at room temperature for 30 min. Plates were then washed with washing buffer and incubated with mouse anti-influenza A NP mAb (in house) for 1 h at room temperature. Plates were washed again with washing buffer then incubated with HRP-conjugated goat anti-mouse IgG for 1 h at room temperature. Plates were washed a final time before substrate solution was added, followed by 0.1 M H_2_SO_4_ to stop the reaction. The plate was read at 450 nm and absorbance values for sample virus were compared to a purified SDS-PAGE quantified control virus (X-31). Absorbance values were plotted against Log_10_[virus] using Graphpad Prism. A curve was fitted using the sigmoidal dose response (variable slope) function. The Log_10_[virus] of sample virus was determined by interpolating absorbance values using the X-31 standard. Binding of purified virus to biotinylated sialic acid receptor analogues, α2,6-sialyllactosamine (6SLN), α2,3-sialyllactosamine (3SLN), and Neu5Ac α2,3 gal β1–4(6-HSO3) GlcNAc (3SLN(6Su)) (GlycoNZ, Auckland, New Zealand), was measured by biolayer interferometry using an Octet RED instrument (Sartorius), and established methods [34]. Streptavidin-coated biosensors (Sartorius) were used to immobilise biotinylated receptor analogues at a concentration range of 0.01 to 0.5 µg ml^−1^ in 10 mM HEPES, pH 7.4, 150 mM NaCl, 3 mM EDTA and 0.005 % Tween-20 (HBS-EP). Virus was diluted to a concentration of 100 pM in HBS-EP consisting of 10 µM oseltamavir carboxylate (Roche, Welwyn Garden City, UK) and zanamivir (Sigma-Aldrich, Gillingham, UK). Association between virus and receptors was measured over 30 min at 20 °C, and the relative amount of virus bound to the biosensor via the receptor analogues at the range of receptor analogue concentrations was calculated using the amplitude of the response at the end of the association step.

### Immunohistochemistry and flow cytometry

For immunohistochemistry (IHC), 293 T cells were transfected with 500 ng 50–92 HA pCAGGS plasmid on glass coverslips and incubated for 24 h. Cells were fixed in 4 % paraformaldehyde in PBS and probed with sheep α-HA antibody (1 : 300) (Vietnam/04, NIBSC) and HA indirectly detected by goat α-sheep antibody conjugated to FITC (1 : 500) (Merck Millipore). Pictures of fluorescent cells were acquired with an Axiovert 200M microscope (Zeiss) with a Axiocam HRC camera (Zeiss) and were edited with AxioVision Rel 4.7 programme. For flow cytometry, HeLa cells in 12-well plate format were transfected with 500 ng of pCAGGS plasmid expressing 50–92 HA and incubated for 24 h. After 24 h incubation, cell supernatants were removed and cells were trypsinised, pelleted by brief centrifugation in 1.5 ml Eppendorf tubes, and resuspended and washed in ice-cold PBS then probed with chicken polyclonal anti-50–92 HA sera (1 : 100) (APHA, Weybridge). HA was indirectly detected by AlexaFlour 488 FITC antibody (1 : 500). Prior to flow cytometry analysis, cells were fixed with 1 % paraformaldehyde in PBS for 10 min on ice. Cells were analysed using a MACSQuant flow cytometer (Miltenyi biotec) and data analysed using FACS Quant software.

## Results

### Mapping amino acid substitutions on the H5 haemagglutinin crystal structure

**Fig. 1.**
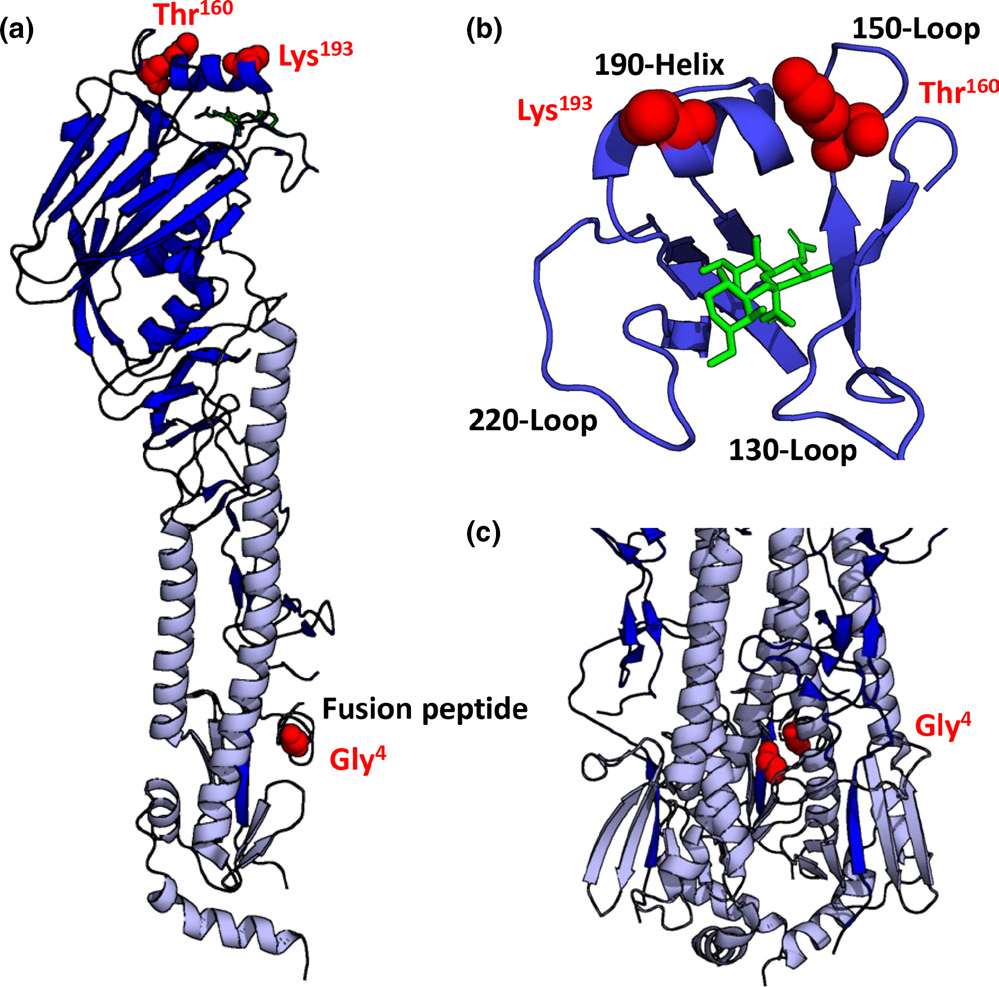
Location of amino acid substitutions on crystal structure of H5 HA. The three amino acid residues identified to differ between LP 50–92 and HP 50–92 were visualised on the crystal structure of H5 HA (PDB: 5E34) [55] using PyMol [56]. (**a**) Cartoon representation of H5 HA monomer with HA1 shown in blue and HA2 shown in pale blue. Residues 160, 193 and HA_2_4 are shown in red as sphere representations. (**b**) The head domain of HA1 is shown in blue with components of the receptor binding pocket, the 130-Loop, 150-Loop, 190-Helix and 220-Loop. A receptor analogue (LSTa) of 2,3 sialic acid is shown in green in the receptor binding pocket. (**c**) A trimer of the HA protein is shown, with a focus on the fusion peptide.

The three HA amino acid residues found to be mutated between LP and HP 50–92 virus were mapped onto the crystal structure of an H5 HA to determine their proximity to important functional regions of HA (Fig. 1). Residues 160 and 193 are located on the head of HA1 close to the receptor binding pocket formed by the 130-loop, 150-loop, 190-helix and 220-loop (Fig. 1b). Residue 160 is adjacent to the prominent 150-loop while residue 193 is adjacent to the 190-helix at the rim of the receptor binding pocket. Residue HA_2_4 is located on the fusion peptide (Fig. 1a, b). The identified amino acid residues in the head domain of HA are exposed where they can interact with their local environment whereas the fusion peptide amino acid residue is partially occluded within the trimer interface of the HA protein (Fig. 1c). The amino acid substitution at residue 160 from alanine (A) to threonine (T) introduces an N-linked glycosylation motif; the resulting glycan at residue 158 would therefore protrude from the 150-loop and be close to the receptor binding pocket. Glycosylation of H5N1 HA residue 158 has been confirmed previously [35].

### Generating HP 50-92 reverse genetics virus

To study the contribution of the identified HA amino acid substitutions to the phenotype change in the highly pathogenic 50–92 virus, we used reverse genetics to generate recombinant wild-type (WT) 50–92 virus [26] with changes introduced into the HA using site-directed mutagenesis. Virus containing single substitutions at residue 160 or 193 could not be generated despite multiple attempts indicating that in isolation these substitutions are detrimental to virus biology. Amino acid substitutions A160T, glutamate (E) to lysine (K) at residue 193 and glycine (G) to arginine (R) at residue HA_2_4 were introduced. The result was an isogenic HA relative to a highly pathogenic 50–92 clone described by [24]. We also generated viruses carrying various combinations of the amino acid identities; thus, viruses containing A160/E193/HA_2_G4 (50–92), A160T/E193K/HA_2_G4R (50–92-HP), A160T/E193K (50-92_160T,193K_), A160T/HA_2_G4R (50-92_160T,4R_), E193K/HA_2_G4R (50-92_193K,4R_) and HA_2_G4R (50-92_4R_) were generated. All viruses retained their MBCS in HA and the remaining gene segments were from wild-type 50–92.

### Small differences in virus replication detected between 50-92 variant viruses in avian cells

**Fig. 2.**
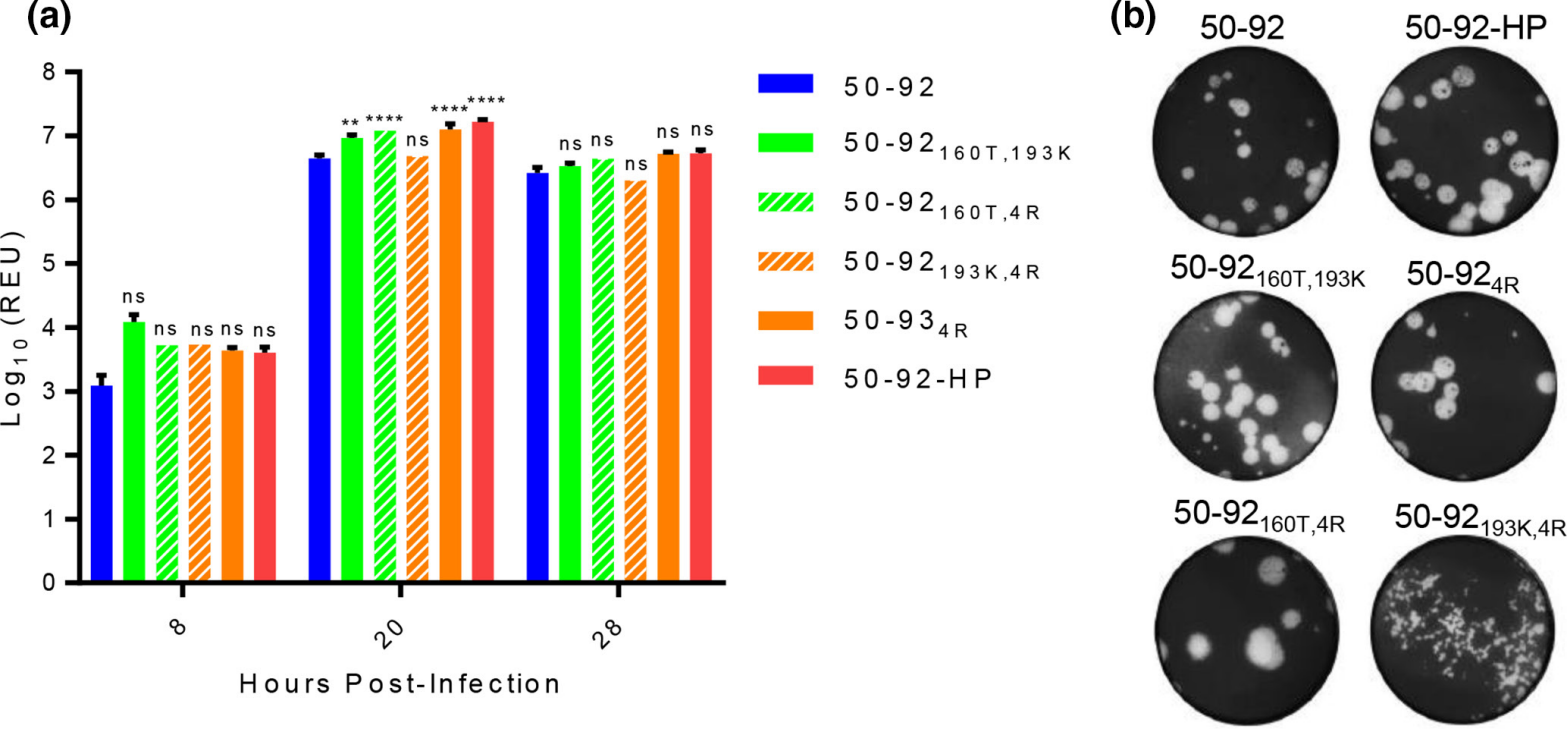
Virus replication kinetics and plaque morphology of 50–92 H5 viruses. (**a**) CEF cells were infected with 50–92 H5N1 viruses (MOI 0.0001), incubated at 41 °C and cell supernatants harvested at 8, 20 and 28 h post-infection. vRNA was extracted from cell supernatants and quantified by qRT-PCR of M gene (REU=Relative EID_50_ Unit). Error bars are SEM. Statistical analysis by two-way ANOVA with Dunnett’s multiple comparisons test. ns=not significant, **P<0.001, ****P<0.00001. (**b**) Plaque assay of MDCK cells infected with a serial dilution of H5N1 virus in the absence of TCPK trypsin. Cells were fixed, stained and imaged using a flatbed scanner.

To determine the impact of the HA amino acid substitutions on virus replication we performed *in vitro* multi-cycle virus replication kinetics experiments in primary chicken embryonic fibroblast (CEF) cells using each of the 50–92 viruses generated above. At 8 and 28 h post-infection there were no statistically significant differences between viral RNA detected in cell supernatants. However, at 20 h post-infection all viruses apart from 50-92_193K,4R_ demonstrated small but statistically significantly increased levels of viral RNA compared to 50–92 (Fig. 2a). Interestingly, E193K enhanced virus replication when in combination with A160T but not HA_2_G4R alone, which suggests an optimal combination of substitutions affected virus replication. As seen with the 50–92 clones described by Wood *et al.*, virus plaque morphologies differed between 50–92 variants (Fig. 2b). The virus 50-92_193K,4R_ showed strikingly small pin-prick virus plaques despite a comparatively modest reduction in vRNA titres when compared to the other 50–92 viruses (Fig. 2b).

### Contribution of A160T, E193K and HA_2_G4R to a highly pathogenic phenotype in chickens

**Fig. 3.**
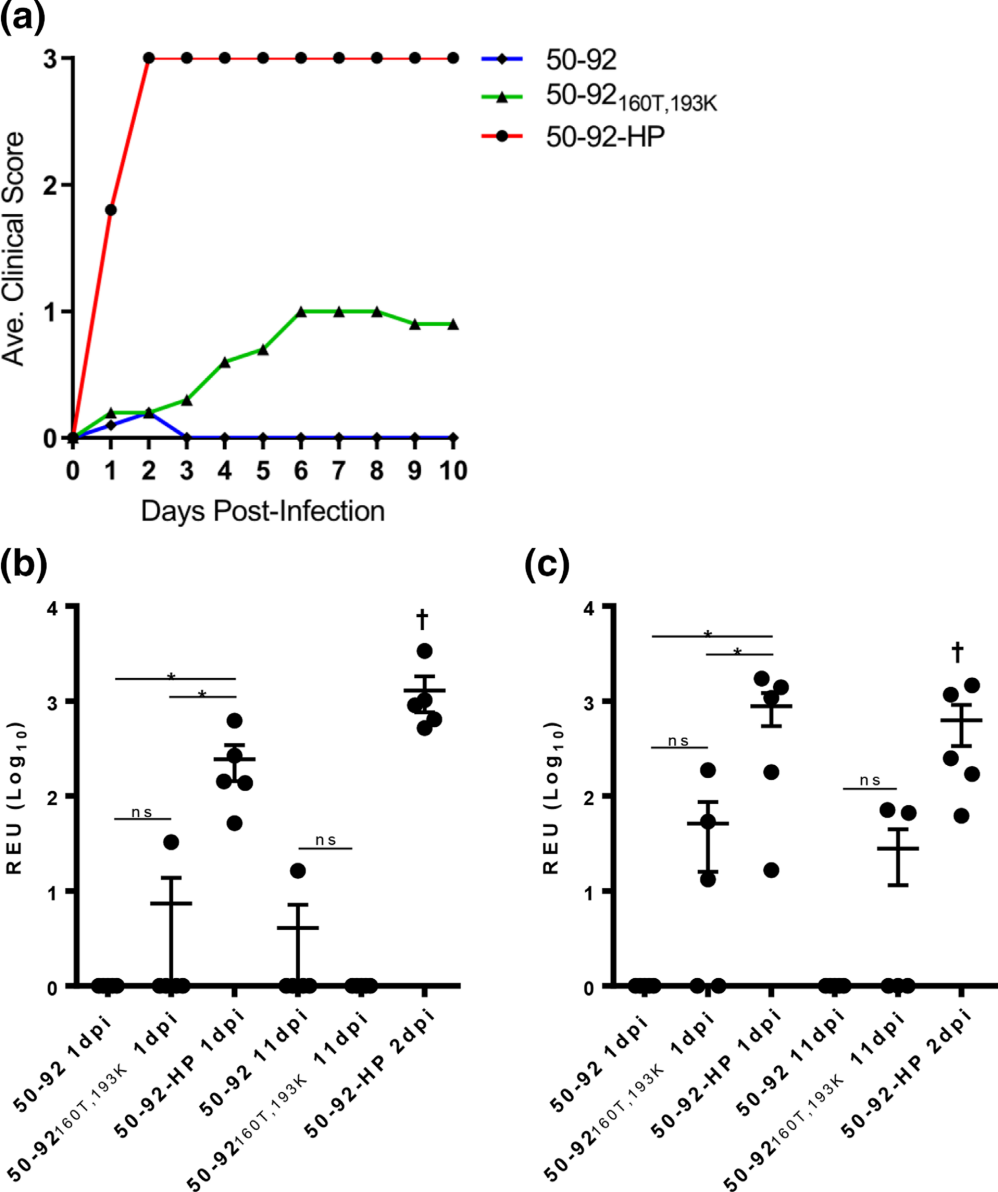
HA substitutions A160T, E193K and HA_2_G4R were required for highly pathogenic phenotype of H5N1 50–92 virus in chickens. IVPI testing was performed for 50–92, 50–92-HP and 50-92_160T,193K_ virus where ten 6 week old SPF chickens were intravenously injected with >16 HA units of virus. (**a**) Birds were scored for clinical signs throughout 10 days (0=normal, 1=sick, 2=severely sick, 3=dead). The 50–92 data points are shown as circles, 50–92-HP as triangles, and 50-92_160T,193K_ as diamonds. Oropharyngeal (**b**) and cloacal (**c**) swabs were taken from five randomly selected birds 1 day post-infection (dpi) and 11 dpi († except 50–92-HP where birds succumbed to infection at 2 dpi). Viral RNA was extracted and quantified by qRT-PCR of M gene (REU=Relative EID_50_ Unit). Error bars are SEM. Statistical analysis of 1 dpi by one-way ANOVA with Tukey’s multiple comparisons test, 11 dpi by unpaired two-tailed *t*-test. ns=not significant, *P<0.01.

Next, we wanted to determine virus pathogenicity in 6 week old chickens using the IVPI test. We were able to test 50–92, 50-92_160T,193K_ and 50–92-HP. These three viruses gave IVPI scores of 0.02, 0.68 and 2.88, respectively. Thus, only 50–92-HP containing the A160T, E193K and HA_2_G4R substitutions maintained a HP phenotype as defined by the IVPI test. The virus 50-92_160T,193K_ led to a greater number of clinical signs in chickens compared to 50–92, which peaked at day six post-inoculation, although no birds succumbed to the infection (Fig. 3a). There was limited viral RNA detected for both 50–92 and 50-92_160T,193K_, although some viral RNA was detected in cloacal swabs from several birds infected with 50-92_160T,193K_ virus (Fig. 3b, c). The 50–92-HP viral RNA was detected in all birds by oropharyngeal and cloacal swabbing day one post-infection at significantly higher levels compared to 50–92 and 50-92_160T,193K_, together with the most severe clinical signs, with all birds succumbing to infection by day two post-infection.

### Amino acid substitution HA_2_G4R increases the pH of fusion

**Fig. 4.**
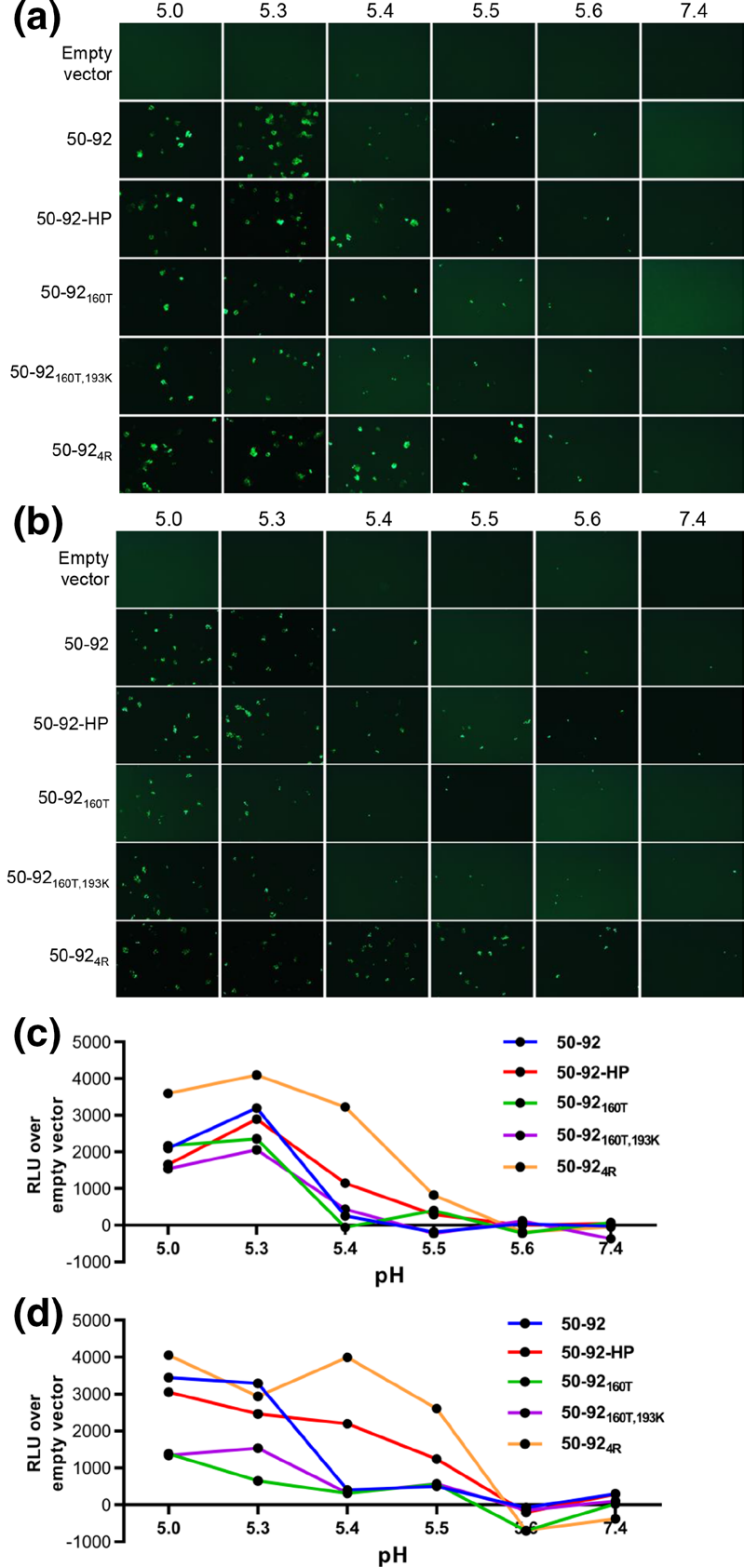
Substitution HA_2_G4R decreased pH of fusion of H5 HA expressed in human 293T and chicken DF-1 cells. Split venus fluorescent HA fusion assays were conducted in 6-well plates of 293 T cells (**a**) or DF-1 cells (**b**). Cells were transfected with 1250 ng pCAGGS 50–92 HA or empty vector together with 1250 ng pBiFC-bJunVN155- or 1250 ng pBiFC-bFosVC155 alone and after 3 hours cell populations were mixed and split into 96-well plates in a 1 : 1 ratio. After 24 hours cells were treated with MES pH buffer at the indicated pH at 37 °C for 5 min before cell media was replaced. After 3 h syncytia were imaged by fluorescence microscopy. Fluorescence intensity of 293 T cells (**c**) and DF-1 cells (**d**) was measured using a plate reader (RLU=relative light unit).

Due to the location of the HA_2_G4R amino acid substitution on the fusion peptide we hypothesised that this may alter the pH of fusion and could account for the increased pathogenesis in chickens. To this end, we employed a bimolecular fluorescence complementation assay to measure HA cell to cell fusion in human 293T and chicken DF-1 cells comparing the impact on pH of fusion by the HA substitutions. Each of the 50–92 HAs were cloned into pCAGGS expression plasmids and transfected into cells along with the bZIP domain containing Jun protein tagged with the N-terminal half of Venus or with Fos tagged with the C-terminal half of Venus protein alone (Fig. S1). At 3 h post-transfection the two cell populations were mixed and incubated for 24 h before being treated with MES pH buffers to activate HA fusion. Successful HA fusion of HA at the cell surface resulted in syncytia formation that permitted the mixing of Jun and Fos and the subsequent complementation of Venus protein that was imaged by fluorescence microscopy (Fig. 4a, b). Fluorescence intensity was measured using a plate reader (Fig. 4c, d). The virus 50-92_4R_ HA displayed the highest pH of fusion with appreciably more fusion seen at pH 5.5 in both 293T and DF-1 cells compared to any other HA, although this was more pronounced in DF-1 cells (Fig. 4). WT 50–92, 50-92_160T_ and 50-92_160T,193K_ all displayed a peak in fusion at pH between 5.0 and 5.3 in both cell types with a dramatic drop in fusion by pH 5.4. The 50–92-HP showed an intermediate pH of fusion with a pronounced increase in DF-1 cells whereby there was appreciable fusion at pH 5.5, albeit to a lesser extent than 50–92 with the HA_2_G4R substitution alone. Both 50-92_160T_ and 50-92_160T,193K_ HAs caused less intense fluorescence in DF1 cells compared to WT 50–92 or HAs containing the HA_2_G4R substitution. Taken together these data suggest the two substitutions close to the receptor binding pocket, A160T and E193K, reduce the pH of fusion and the substitution in the fusion peptide increases the pH of fusion (Fig. 4).

### Amino acid substitution HA_2_G4R reduces pH stability

**Fig. 5.**
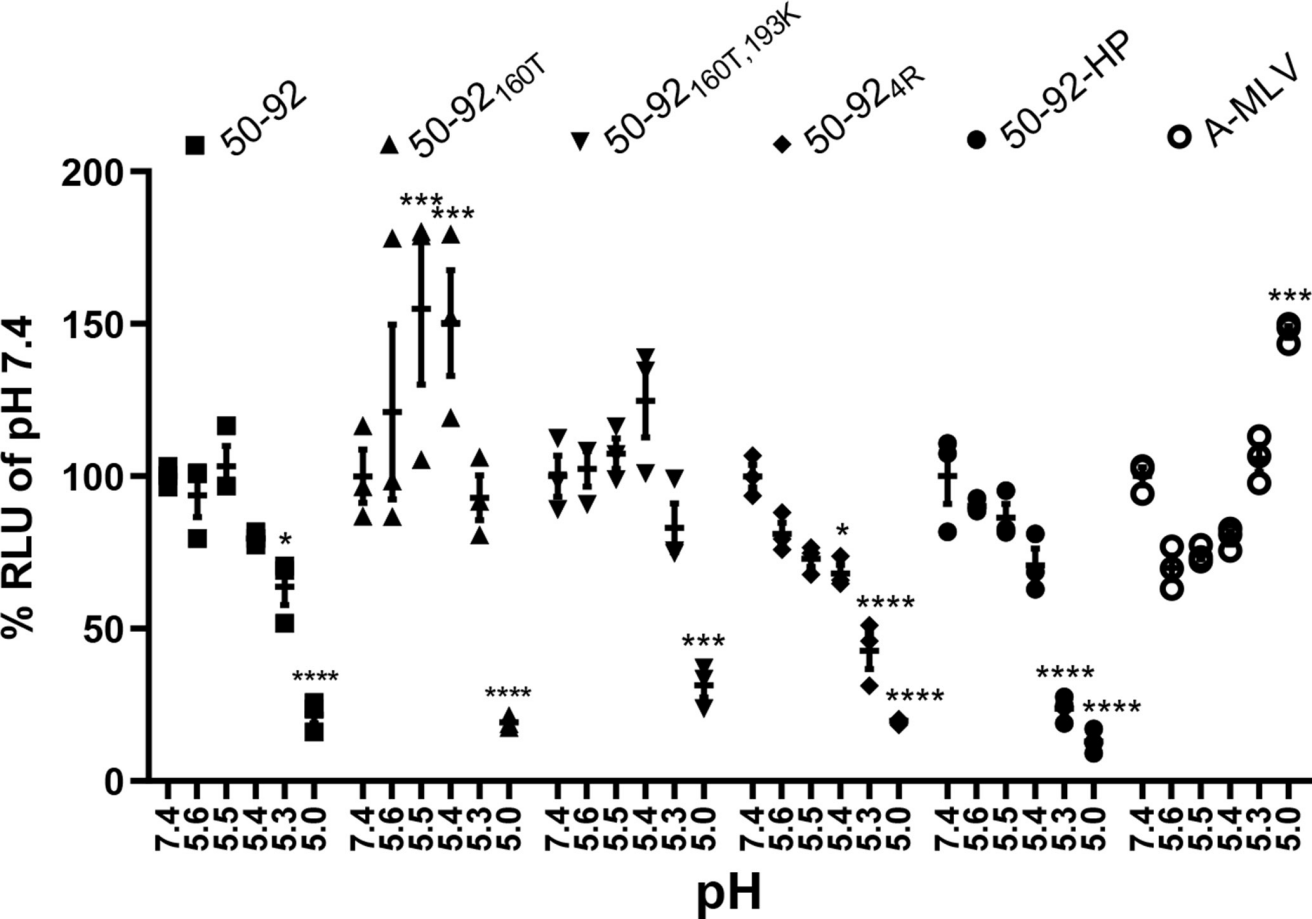
Substitution HA_2_G4R decreased pH stability of H5 pseudotypes. Infection of HEK 293 T cells with 50–92 H5 pseudotypes encoding firefly luciferase. Either 50–92, 50-92_160T_, 50-92_160T/193K_, 50-92_4R_, 50–92-HP VLPs were mixed with an equal volume of MES pH buffer (7.4, 5.6, 5.5, 5.4, 5.3, 5.0) at 37 °C for 5 min and inoculated onto 293 T cells in 96-well plates in triplicate. A-MLV pseudotype was treated similarly, as a pH independent entry control. After 24 hours cells were lysed in passive lysis buffer and firefly luminescence quantified and measured by a plate reader. Data were normalised to % relative light unit (RLU) of VLPs treated at pH 7.4. Data points are shown as squares for 50–92, upward triangles for 50-92_160T_, downward triangles for 50-92_160T, 193K_, diamonds for 50-92_4R_, filled circles for 50–92-HP, and empty circles for the A-MLV control. Statistical analysis by two-way ANOVA with Dunnett’s multiple comparisons test, ns=not significant, *P<0.1, ***P<0.001, ****P<0.0001.

To extend our studies on the impact of HA_2_G4R on pH of fusion we conducted pH stability assays using influenza PVs, as a safer alternative to HPAIVs [32]. The PVs were treated with MES buffers at various pH values and inoculated onto 293 T cells. The rationale was that PVs with reduced pH stability would have reduced capacity to infect cells due to premature fusion-activation, quantified by measuring luciferase expression from successfully transduced PVs. A PV was generated using the envelope protein of amphotropic murine leukaemia virus (A-MLV), whose cell entry is pH-independent, and was used as a control. The two PV containing the HA_2_G4R substitution, 50–92-HP and 50-92_4R_, had significantly reduced capacity to infect cells at >pH 5.4 whereas all other viruses did not, suggesting these PV were less pH stable (Fig. 5). Interestingly, 50-92_160T_ significantly increased its capacity to infect cells at >pH 5.4. These data corroborate what was seen with the pH of fusion data (Fig. 4) whereby A160T and E193K in the receptor binding pocket increase pH stability and HA_2_G4R on the fusion peptide reduce pH stability; together these substitutions optimise pH stability. We confirmed that each substitution studied did not alter the pCAGGS driven expression of HA in cells (Fig. S1, available in the online version of this article).

### Amino acid substitutions A160T and E193K modulate receptor binding

**Fig. 6.**
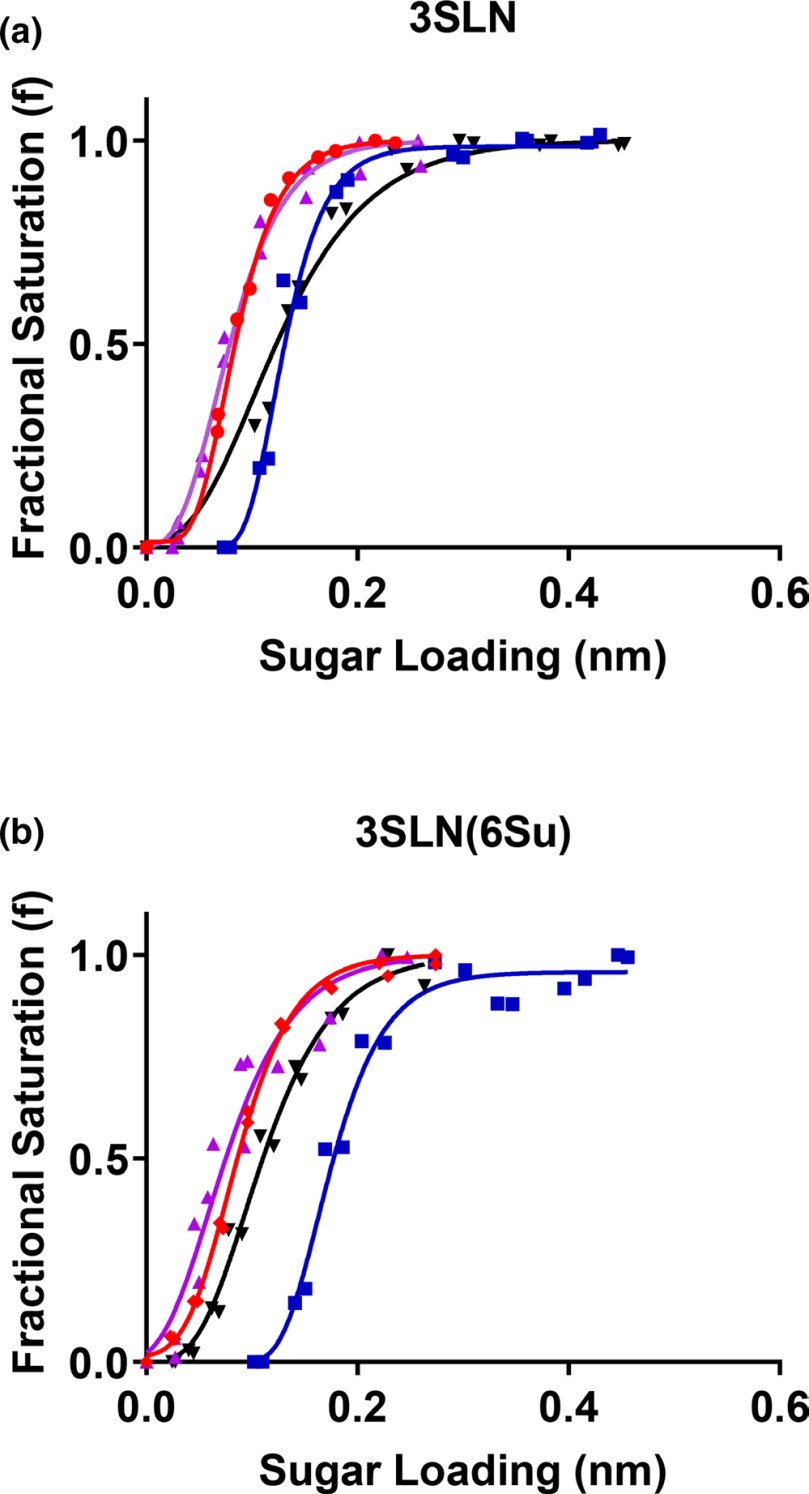
Receptor binding profiles of H5N1 50–92 virus. Biolayer interferometry was used to determine the impact on receptor binding of three amino acid substitutions in the HA protein. The impact of substitutions was determined for both α2,3-sialyllactosamine (3SLN) (**a**) and Neu5Ac α2,3 Gal β1-4(6-HSO3)GlcNAc (3SLN(6su)) (**b**). Blue lines with squares show binding of sb50-92, red circles of sb50-92_160T,193K_, purple upward triangles of sb50-92_E193K_, and black downward triangles of sb50-92_160T_. There was no detectable binding to the receptor analogue α2,6-sialyllactosamine (6SLN).

Due to the proximity of the A160T and E193K amino acid substitutions to the receptor binding pocket we wanted to investigate their impact on virus receptor binding. To this end we reconstructed the reverse genetics 50–92 viruses to exclude the MBCS and to contain H1N1 PR8 internal genes in order to conduct biolayer interferometry. These viruses were named single-basic 50–92 (sb50-92). Interestingly, by taking this approach we were able to generate viruses with the single substitutions of A160T and E193K in addition to the A160T/E193K double mutant. These sb50-92 viruses (including sb50-92 with A160 and E193 residues) were purified and used in biolayer interferometry to assay receptor binding phenotype. We assayed receptor binding against avian-like 3′-sialyllactosamine (3SLN), human-like 6′-sialyllactosamine (6SLN) and a sulphated version of the avian-like 3SLN called 3SLN(6Su), which are commonly used analogues of host cell 2,3 and 2,6 sialic acid (SA) [36, 34]. When taken in isolation, the E193K substitution had the largest impact on receptor binding, facilitating a 46-fold increase in binding to 3SLN(6Su) and a six-fold increase in binding to 3SLN relative to sb50-92 with A160 and E193 (Fig. 6, Table 1). Furthermore, in isolation the A160T substitution facilitated an eight-fold increase in binding to 3SLN(6Su) but a three-fold decrease in binding to the non-sulphated analogue, 3SLN (Fig. 6, Table 1). Together, the A160T/E193K substitutions facilitated a 23-fold increase in binding to 3SLN(6Su), and an eight-fold increase in binding to 3SLN (Fig. 6, Table 1). The reduced binding of A160T/E193K to 3SLN(6Su) relative to E193K indicates the addition of an N-linked glycan at residue 158 optimizes, by decreasing, the significant increase in binding facilitated by E193K alone. There was no detectable binding to 6SLN by any of the viruses tested.

**Table 1.** Fold-changes in receptor binding

	Fold-change binding per sugar	
H5N1 50–92 variants	3SLN(6Su)	3SLN
**sb50-92 (A160, E193, G4)**	na	na
**sb50-92_160T_**	8× increase	3× decrease
**sb50-92_193K_**	46× increase	6× increase
**sb50-92_160T,193K_**	23× increase	8× increase

### Natural occurrence of identified substitutions

Finally, we wanted to determine the natural occurrence of each amino acid at residues 160, 193 and HA_2_4 studied here. We also extended our analysis to include residues 158 and 159 to incorporate the full N-linked glycosylation motif associated with residue 160 (Table 2). For avian H5N1 HA, 61 % of sequences contained an N and 36 % contained a D at residue 158. Furthermore, at HA 160, 63 % of sequences contained an A, 35 % a T and <1 % an S (Table 2). Taken together 32 % (*n*=975) of avian H5N1 HA sequences carry a potential N-linked glycosylation motif between residues 158 and 160. For human H5N1 HA, 81 % of sequences contained an N and 17 % a D. At HA 160, 34 % of sequences contained an A, 63 % a T and 1.6 % an S (Table 3). Taken together 64 % (*n*=277) of human H5N1 HA sequences carry a potential N-linked glycosylation motif between residues 158 and 160. We found HA 193 was highly conserved with a preference for a positively charged amino acid, K (53 and 59 %; human and avian) or R (44 and 34 %; human and avian), suggesting a selective pressure exists for a positively charged amino acid in H5N1 HA protein at this residue. We identified total conservation of G at HA_2_4 in human H5N1 viruses and almost total conservation in avian H5N1 viruses; there was one virus with A and one virus with R at HA_2_4.

**Table 2.** Percentage frequency of amino acids at the studied residues for avian H5N1 viruses Full-length nucleotide sequences of H5N1 avian (*n*=2997) were downloaded from the NCBI database and analysed to determine the natural occurrence of each amino acid at residues 158, 159, 160, 193 and HA_2_4.

		Position				
		158	159	160	193	HA_2_4
**Amino acid**	**N**	61.90	62.90	–	1.07	–
	**D**	36.70	15.68	–	0.70	–
	**R**	–	–	–	59.03	0.03
	**A**	–	0.40	63.26	–	0.03
	**Q**	–	–	–	0.57	–
	**E**	0.03	–	0.03	0.23	–
	**G**	1.00	0.30	–	0.30	99.93
	**H**	–	0.03	–	–	–
	**I**	–	–	0.13	–	–
	**K**	–	–	0.03	34.80	–
	**M**	–	–	0.13	2.37	–
	**S**	0.37	20.62	0.73	0.10	–
	**T**	–	0.07	35.67	0.83	–

**Table 3.** Percentage frequency of amino acids at the studied residues for human H5N1 viruses Full-length nucleotide sequences of H5N1 human (*n*=427) HA genes were downloaded and analysed as above

		Position				
		158	159	160	193	HA_2_4
**Amino acid**	**N**	81.50	47.54	–	0.23	–
	**D**	17.10	5.62	–	–	–
	**R**	–	0.23	–	53.86	–
	**A**	–	–	34.66	–	–
	**E**	0.23	–	–	0.47	–
	**G**	0.94	–	–	0.23	100.00
	**K**	–	–	–	44.73	–
	**S**	–	46.37	1.64	0.23	–
	**T**	0.23	0.23	63.70	0.23	–

## Discussion

We sought to examine which of the three HA amino acid differences in 50–92 HA were responsible for a change in virus pathogenesis. We found all three HA substitutions were necessary for the HP phenotype and increased virus shedding in chickens. Reverse genetics generated WT 50–92 was severely attenuated with negligible shedding detected *in vivo*, suggesting the original consensus sequence of the virus isolate from this HP outbreak in turkeys did not account for the pathogenic phenotype, and the clones discovered in the original quasispecies must have been responsible for the observed pathogenesis [24]. The combination of 160T and 193K alone failed to confer the HP phenotype in chickens, although there was some evidence that they might have contributed to increased replication *in vivo*. Substitution HA_2_G4R was necessary to complete the transformation into an HPAIV. We did not determine whether HA_2_G4R was capable of the HP phenotype alone, and whether substitutions 160T and 193K were indeed required for the HP phenotype. However, SA binding analysis together with bioinformatic analysis suggests there is a strong preference for a charged amino acid at residue 193 as is typically found in H5N1 viruses.

H5N1 50–92 carries a short-stalk (ss) NA due to a 22aa deletion which could be linked to the need for a glycan at residue 158. HA lacking a glycan at 158 restricts virus growth when combined with a ssNA [37]. When a long-stalk NA is combined with a glycosylated 158 there is poor virus growth. Intriguingly, for 50–92 virus, the 193K substitution was detrimental for 50–92 virus in combination with HA_2_G4R alone, resulting in a small plaque size and reduced growth compared to 50–92-HP. This is likely due to reduced virus release, since 193K significantly increases binding, and the lack of a glycosylation site to sterically block SA binding results in an ‘overactive’ HA. When 160T is added alongside 193K the dramatic increase in binding avidity driven by 193K alone is somewhat ameliorated by the additional glycan. The contribution of N-linked glycans in the HA protein to pathogenesis has been studied for several HP viruses. In an H7N7 virus, the addition of a glycan to the head region of HA1 was shown to recover the HP phenotype in chickens compared to an LP variant that lacked the glycosylation site [38].

Interestingly, neither 160T nor 193K, nor combinations thereof facilitated virus binding to human-like 2,6 SLN. Despite this, amino acid substitutions close to the receptor binding pocket of H5N1 HA have previously been shown to promote human-like 2,6 SLN binding [39, 40]. We also confirmed the importance of the sulphated 2,3 sialic acid analogues to AIVs of the H5N1 subtype; all viruses tested here showed receptor binding to both non-sulphated and sulphated analogues. This suggests that the 160T and 193K in the context of this H5N1 virus does not heighten the risk of zoonotic infection and in fact reinforces poultry adaptation when taking into account the HA_2_4R substitution [41–44]. It is likely these substitutions are required for efficient viral binding and release in the chicken host.

Bioinformatic analysis highlighted the novelty of the HA_2_G4R substitution; almost all H5N1 viruses contain a non-polar G at HA_2_4 whereas the HA_2_G4R amino acid substitution studied here represents a swap of G for positively charged R. We are not aware of previous descriptions of the effects of the HA_2_G4R substitution in the literature. Instead a G4A substitution has been studied and shown to increase the pH of fusion by 0.4 pH units [45]. In addition, removal of the negative charge in the fusion peptide has been shown to result in deeper penetration of the fusion peptide into the host cell membrane [46]. This observation may suggest that the HA_2_G4R substitution could result in deeper penetration of the fusion peptide, allowing fusion to occur at a higher pH. Introducing either 160T or 193K resulted in a reduced pH of fusion compared to WT 50–92. The pH of fusion was recovered by the HA_2_G4R substitution, to a level more usual for avian viruses, around pH 5.5 [43, 47–49].

Receptor binding mutations have previously been described to alter the stability of the HA. The H5 HA of Vietnam/04 was adapted for ferret respiratory droplet transmission by Imai *et al.* [50]. The introduction of receptor binding mutations N224K/Q226L increased the pH of fusion compared to WT HA. Removal of a glycosylation site, N158D, did not appear to alter pH of fusion further. To compensate this change in pH a fourth mutation was selected, T318I which stabilized the HA, and decreased pH of fusion either alone or in combination with the receptor binding mutations. Since human viruses have been shown to require a lower pH of fusion than avian-restricted AIVs, the T318I mutation was required to correct the negative effect of the receptor switching mutations. Previously, a similar set of mutations described a HA mutation likely to alter pH of fusion, H103Y, although this was not confirmed [51]. Clearly all three sets of mutations were required together to adapt 50–92 HA for increased pathogenicity in poultry, and the function described by each of these mutations is remarkably like those described for the two H5 viruses that were adapted to ferrets.

Likewise, a previous study found that H5 H24Q substitution decreased pH of fusion to promote efficient transmission in ferrets when coupled with receptor binding changes and a loss of an N-linked glycan at residue 158 [52]. Thus, H5 viruses offer an interesting model for studying virus mechanisms of pathogenesis beyond the MBCS and understanding the interplay of pathogenesis, species-specificity, and transmission.

The pH of HA fusion has previously been linked to pathogenesis in the avian host. A panel of H5N1 viruses that ranged in their pH fusion demonstrated an increase in pathogenicity in chickens that correlated with an increase in the pH of fusion [47]. Similar experiments in ducks described an optimal pH of fusion that when decreased resulted in decreased pathogenicity, but when increased further the unstable HA was detrimental to virus transmission [49]. This observation prompted the intriguing hypothesis that selection of unstable HAs may increase pathogenesis but at the cost of efficient transmission, whereby such AIVs may not be spread further via wild birds [53]. The mechanism underlying the increase of pathogenesis by a pH unstable HA remains to be understood, although the potent host factor Interferon-Inducible Transmembrane Protein 3 (IFITM3) has been suggested as a possible driver for this adaptation. Chicken IFITM3 prevents virus entry by blocking virus HA fusion with the late endosomal membranes and it is suggested that viruses may evade this factor by fusing at a higher pH in the earlier endosome membranes [54].

The amino acid substitutions described by this work highlight a route to increased pathogenicity by mutations accumulating in HA including a novel mutation that affects pH of fusion. In summary, these data provide useful information for understanding virulence mechanisms of H5N1 viruses for poultry and contribute to the concept of pH as a virulence factor and interspecies host barrier for influenza virus.

## Supplementary Data

Supplementary material 1
