# Supplementary material for: Amino acid substitutions in the H5N1 avian influenza haemagglutinin alter pH of fusion and receptor binding to promote a highly pathogenic phenotype in chickens
Source: J Gen Virol. 2021 Nov 2;102(11):001672. doi: 10.1099/jgv.0.001672 (PMC8742987; doi:10.1099/jgv.0.001672)
Supplement: Supplementary material 1 [file jgv-102-1672-s001.pdf]

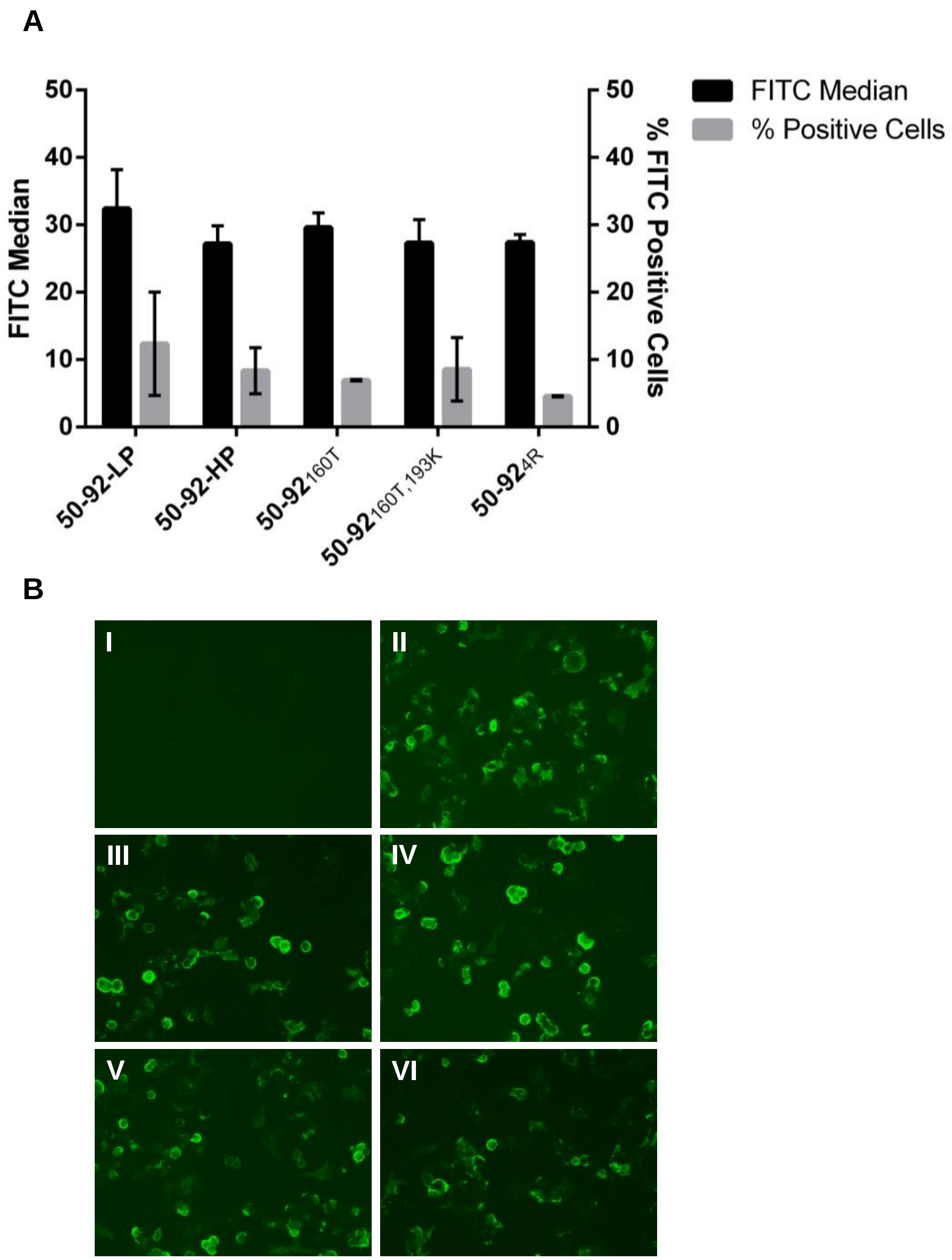

Supplementary figure 1. Expression of H5 HA mutants in human 293T and HeLa cells

(A) HeLa cells were transfected with 500ng of pCAGGS expression plasmids containing 50-92 HAs in 12-well plate format, plus empty vector mock. After 24hrs cells were washed and probed with chicken polyclonal anti-50-92-HA sera (1:100) (APHA, Weybridge). HA was indirectly detected by AlexaFlour 488 FITC antibody (1:500). Cells were fixed in 4% paraformaldehyde before flow cytometry was performed. Data from two independent experiments is displayed here as median fluorescence with percentage FITC positive cells. Error bars are SEM. Statistical analysis by two-way ANOVA revealed no significant differences. (B) 293T cells were transfected as above but on glass coverslips. After 24hrs cells were fixed in paraformaldehyde. Cells were incubated with sheep α-HA antibody (1:300) (Vietnam/04, NIBSC) and HA indirectly detected by goat α-sheep antibody conjugated to FITC (1:500) (Merck Millipore). (I) Empty Vector, (II) 50-92, (III) 50-92-HP, (IV) 50-92$_{160T}$, (V) 50-92$_{160T/193K}$, (VI) 50-92$_{4R}$, X20 magnification.
